# DNA damage and cell cycle events implicate cerebellar dentate nucleus neurons as targets of Alzheimer's disease

**DOI:** 10.1186/1750-1326-5-60

**Published:** 2010-12-20

**Authors:** Jianmin Chen, Mark L Cohen, Alan J Lerner, Yan Yang, Karl Herrup

**Affiliations:** 1 Dept. of Cell Biology and Neuroscience, Rutgers University, 604 Allison Road, Piscataway, NJ 08854, USA; 2 Dept. of Pathology, Case Western Reserve University School of Medicine, 10900 Euclid Avenue, Cleveland, OH 44106, USA; 3 Dept. of Neurology, Case Western Reserve University School of Medicine, 10900 Euclid Avenue, Cleveland, OH 44106, USA

## Abstract

**Background:**

Although the cerebellum is considered to be predominantly involved in fine motor control, emerging evidence documents its participation in language, impulsive behavior and higher cognitive functions. While the specific connections of the cerebellar deep nuclei (CDN) that are responsible for these functions are still being worked out, their deficiency has been termed "cerebellar cognitive affective syndrome" - a syndrome that bears a striking similarity to many of the symptoms of Alzheimer's disease (AD). Using ectopic cell cycle events and DNA damage markers as indexes of cellular distress, we have explored the neuropathological involvement of the CDN in human AD.

**Results:**

We examined the human cerebellar dentate nucleus in 22 AD cases and 19 controls for the presence of neuronal cell cycle events and DNA damage using immunohistochemistry and fluorescence in situ hybridization. Both techniques revealed several instances of highly significant correlations. By contrast, neither amyloid plaque nor neurofibrillary tangle pathology was detected in this region, consistent with previous reports of human cerebellar pathology. Five cases of early stage AD were examined and while cell cycle and DNA damage markers were well advanced in the hippocampus of all five, few indicators of either cell cycle events (1 case) or a DNA damage response (1 case) were found in CDN. This implies that CDN neurons are most likely affected later in the course of AD. Clinical-pathological correlations revealed that cases with moderate to high levels of cell cycle activity in their CDN are highly likely to show deficits in unorthodox cerebellar functions including speech, language and motor planning.

**Conclusion:**

Our results reveal that the CDN neurons are under cellular stress in AD and suggest that some of the non-motor symptoms found in patients with AD may be partly cerebellar in origin.

## Background

The cerebellum has traditionally been considered to be a brain structure that is primarily involved in the regulation of motor coordination, balance and the motor aspects of speech. This view is changing, however, as recent studies have made it clear that the role of the cerebellum extends to higher cognitive functions including language and executive functioning. The cerebellar hemispheres are substantially enlarged in the human brain and the interaction of their output neurons in the dentate nucleus with higher cortical areas is particularly noteworthy. For a full discussion and list of references the reader is referred to the recent review by Strick et al. [1]. Emphasis on this expanded view of cerebellar function can also be found in the variety of linguistic and behavioral disorders that are found to occur following acquired cerebellar lesions [2]. The common features of these functional deficiencies have been termed "cerebellar cognitive affective syndrome" [3] - a set of symptoms that bears a striking similarity to many of the known symptoms of AD.

Neuronal loss in the cerebellum is part of the normal aging process in human. According to the early study of Hall et al. (1975), the loss of cerebellar Purkinje cells occurs at a rate of 2.5% per decade; the functional consequences of this cerebellar atrophy on neurological functions are unknown [4]. However, the cerebellum is not uniformly affected during the aging process. For example, there is currently no evidence of age-related neuronal loss in the dentate or other cerebellar nuclei [5]. The impact of aging on cerebellum and its multimodal interactions with the rest of the brain led us to question whether the neuropathological changes in this brain region might contribute to the neurological and behavioral symptoms of Alzheimer's disease. The degeneration of Purkinje and granule cells has been reported in both familial and sporadic AD [6,7]. Studies have shown diffuse amyloid plaques in the cerebellar cortex of AD patients. The majority of these deposits occur in the molecular layer, and while they may extend into the Purkinje cell layer they rarely occur in the granule cell layer [8,9]. Ubiquitin-immunoreactive dystrophic neurites, increased microglial density and evidence of astrocytosis are also found in the AD cerebellum [6,10,11]. In the later stages of AD, a reduction of cerebellar glucose metabolism is observed [12]; and classic cerebellar functions such as balance and gait are also affected [13,14]. Despite these observations, the cerebellum is still widely regarded as being spared by Alzheimer's disease - often serving as a control tissue or a reference region in imaging studies.

The cerebellum, through its role in the response to pain and emotion, has been linked to behavioral changes such as impulsive behavior. In patients with AD, secondary behavioral manifestations range from hyperactivity to agitation, and impulsive behaviors are common [15]. Motor impulsivity has also been shown in transgenic mice carrying the human APP gene with the Swedish mutation for familial AD (APPsw mice) [16], providing strong evidence of a direct link between mutant APP and this single behavioral AD phenotype both in human and mouse. Impulsive behavior has been traced to abnormalities in the cerebellar deep nuclei, the output nuclei of the cerebellum [17]. While the deep cerebellar nuclei in AD are largely devoid of amyloid deposits and tau inclusions, quantitative MR phase-corrected imaging has revealed increased iron deposition [18]. Iron is often cited for its role in the production of oxidative damage during the progression of AD, raising the significance of this observation. In addition, the loss of cerebellar dentate neurons has been documented in AD with myoclonus [19]. Taken together, these observations suggest that the deep cerebellar nuclei are plausible targets of AD pathology.

Our studies of the biological causes of neuronal death in AD have been guided by our observation of cell cycle reentry in cellular populations that degenerate in human disease [20-22]. Many other laboratories have reported similar findings (references in Herrup and Yang, 2007 [23]). The value of this perspective has been demonstrated in our analyses of mouse models of AD. While neurodegeneration is rarely seen in mouse, in the R1.40 AD mouse model there is a good correspondence between the sites of unscheduled cell cycle events (CCEs) and the areas subject to neurodegeneration in human AD [24-26].

In addition to ectopic cell cycling, AD is also linked to DNA damage. Accumulation of DNA damage in neurons is associated with aging [27] and is exacerbated in neurodegenerative disorders including AD [28,29]. The correlation of both CCE and DNA damage with the appearance of AD suggests that there may be a link between these two processes. This prompted us to ask whether pathological changes can be found in CDN neurons from AD using early markers of cellular stress. In pursuit of this question, we examined the cerebellar dentate nucleus in 46 AD and control specimens at various disease stages. Our outcome measures included the appearance of DNA damage responses and ectopic cell cycle events revealed by immunohistochemistry and fluorescent in situ hybridization (FISH) analysis of DNA replication. We find that the cerebellar dentate nuclei are indeed involved in the pathogenesis of AD, but that their involvement does not begin until the disease is established. Clinical-pathological correlations reveal that cases with moderate to high levels of cell cycle activity in their CDN are highly likely to show deficits in unorthodox cerebellar functions including speech, language and motor planning. We propose that the symptoms of AD are reasonably ascribed, at least in part, to a loss of cerebellar function.

## Results

### Cell cycle events are elevated in AD cerebellar dentate nuclei

**Figure 1:**
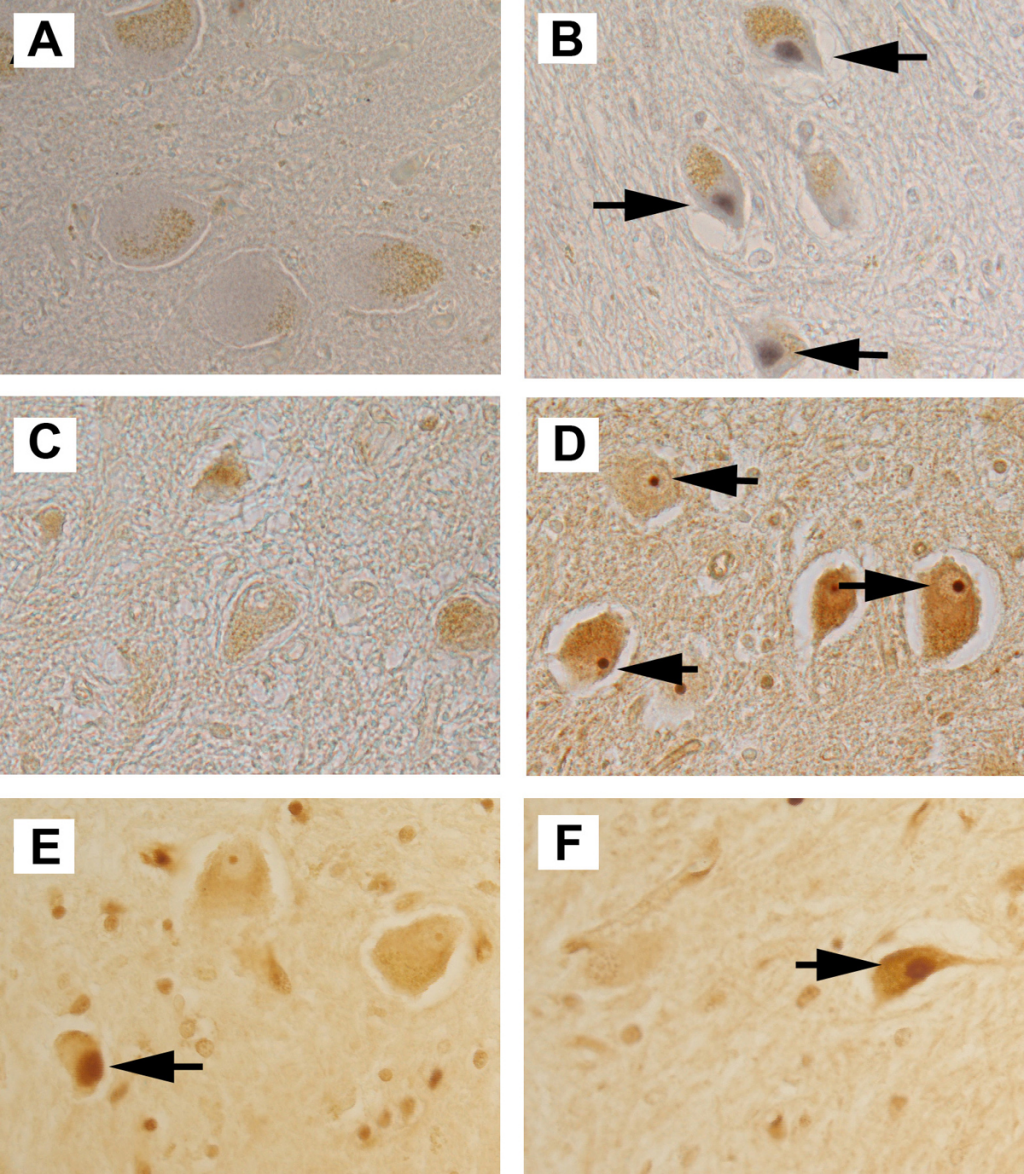
**Cell cycle markers are present in cerebellar dentate nuclear neurons in material from cases with human AD**. Immunostaining for cyclin A, Cdk4 and PCNA are indicated by black arrows in controls (A, C, E) and AD cases (B, D, F). (A-B) Cyclin A (arrows) was found in the nucleus of neurons in the cerebellar dentate nucleus in some AD cases but not controls. The brown material is lipofuscin. (C-D) Strong nucleolar staining of Cdk4, against a background of clear nucleoplasm (arrows), was more prevalent in AD material (D) than in control (C). (E-F) PCNA immunostained neurons were found in the cerebellar dentate nucleus, but controls (E) and AD cases (F) showed no significant difference.

Human cerebellar tissue sections containing both cortex and dentate nucleus from 41 individuals were examined (22 AD; 19 controls). Male to female ratio in both groups were about 1 to 1. Age and post mortem interval (PMI) of AD and control cases were comparable: AD (age = 79 ± 11 years; PMI = 18 ± 17 hours); control (age = 72 ± 19 years; PMI = 19 ± 20 hours). Figure 1 illustrates the type of results we see when we perform immunohistochemistry for cell cycle markers including cyclin A, PCNA and Cdk4. The number of cyclin A positive cerebellar dentate neurons was generally low, but some AD brains contained significant numbers of such neurons (Figure 1B). To provide additional depth to this analysis, we established a rating scale for staining intensity and performed a quantitative comparison based on the average of these scores. As a group, the cerebellar nuclei of AD subjects contained significantly more cyclin A positive neurons than controls (p = 0.0004, Table 1). Importantly, cyclin A expression correlated well with Alzheimer's disease diagnosis and/or with Braak stage (p = 0.0002, Table 2).

**Table 1:** Staining scores for cell cycle and DNA damage markers

Case Number	Diagnoses	Immunocytochemical score				
		
		CycA	PCNA	Cdk4	53BP1	pATM
1	AD	0	1	1	2	-
2	AD	1	-	2	1	-
3	AD	3	2	2	5	4
4	AD	0	0	5	0	0
5	AD	1	0	2	0	5
6	AD	1	0	4	0	3
7	AD	0	1	5	0	3
8	AD	1	1	2	5	4
9	AD	1	1	1	0	-
10	AD	2	0	0	4	5
11	AD	0	1	2	4	5
12	AD	1	0	2	5	5
13	AD	3	3	5	0	5
14	AD	0	1	1	3	5
15	AD	0	1	0	5	-
16	AD	1	1	-	0	-
17	AD	0	0	-	2	-
18	AD	3	3	5	2	0
19	AD	1	0	5	4	5
20	AD	4	3	1	1	1
21	AD	2	1	4	1	3
22	AD	0	0	4	0	5
23	Con	0	0	0	0	0
24	Con	0	0	0	1	0
25	Con	0	2	0	2	0
26	Con	0	2	0	0	0
27	Con	0	0	0	0	0
28	Con	0	1	3	0	4
29	Con	0	0	2	0	-
30	Con	0	0	0	1	0
31	Con	0	0	0	0	0
32	Con	0	0	0	0	0
33	Con	0	0	0	0	0
34	Con	0	0	2	0	0
35	Con	-	2	0	0	0
36	Con	0	3	-	0	0
37	Con	0	2	-	0	0
38	Con	0	2	-	0	0
39	Con	0	2	-	0	0
40	Con	1	1	1	0	0
41	Con	0	1	3	2	0

P value t-test (2-tailed)		CycA	PCNA	Cdk4	53BP1	P-ATM
		
		0.0004	1.0	0.0005	0.001	0.000001

Each sample was scored based on % of CDN neurons stained and the staining intensity. 0 = no staining; 1 = very few cells stained or weakly stained; 2 = moderate staining, 5-10% of the CDN neurons; 3 = 10-25% moderately to strongly stained; 4 = more than 25% strongly stained; 5 = more than 50% strongly stained. The p-values were calculated with Student's t-test, 2-tailed.

**Table 2:** DNA damage responses and cell cycle events correlate with AD disease state

Immunohistochemistry		AD cases	Control cases	Fisher's Exact test p(< 0.05)	Chi-square with Yates correction	
					**κ**^**2**^	p(< 0.05)
**Cyclin A**	+	14	1	0.0002	11.88	0.0006
	-	8	17			
**PCNA**	+	13	8	0.34	0.59	0.44
	-	8	10			
**Cdk4**	+	18	5	0.0009	9.83	0.002
	-	2	10			
**53BP1**	+	14	4	0.01	5.88	0.015
	-	8	15			
**P-ATM**	+	12	1	0.0001	16.1	0.0001
	-	2	15			

Cases marked "+" represent positively stained cases with scores between 1 and 5; cases marked "-" represent those with scores of 0

Cdk4, a G1 cell cycle kinase also revealed a staining pattern that was well correlated with disease. In our material, we found that immunoreactive Cdk4 could be found in two different sub-cellular distributions. In non-demented controls, nuclear Cdk4 could be seen in the neurons of the cerebellar dentate nucleus in 3 of 15 cases. Only 1 out of 20 AD cases showed this pattern in dentate neurons. In the AD cases, however, there were significant numbers of neurons in which the nucleolus was strongly stained for Cdk4 while the remaining nucleoplasm was relatively immunonegative. An example of such a cell is shown in Figure 1D. Cell counts revealed that there were significantly more dentate nucleus neurons with nucleolar Cdk4 in AD brains than in controls (p = 0.0005, Table 1). There was also a robust correlation between nucleolar Cdk4 and AD diagnosis (p = 0.0009, Table 2).

Despite these indications of a reactivation of cell cycle processes in cerebellar dentate nuclei of the AD brain, we found that there was no significant difference (p = 1.0, Table 1) between AD and controls in the number of dentate nuclei immunoreactive for PCNA (proliferating cell nuclear antigen), a component of the DNA replication complex that is elevated during S-phase. Neither was there any correlation between the presence of PCNA staining and AD diagnosis (p = 0.34, Table 2). This discrepancy was unexpected, but led us to consider the fact that PCNA is involved in DNA damage repair [30] as well as in DNA replication.

### DNA damage responses are detected in AD cerebellum

**Figure 2:**
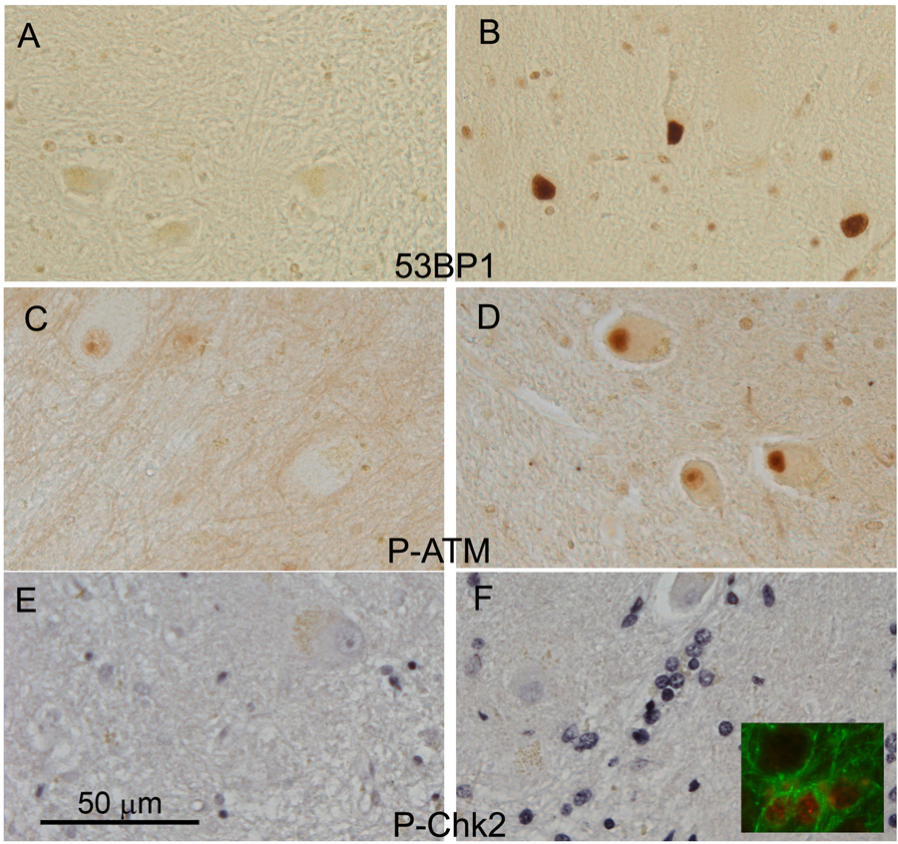
**Evidence of DNA damage can be detected in AD cerebellar deep nuclear neurons and surrounding non-neuronal cells**. (A-B) 53BP1 staining is virtually absent in control material (A) but robust in neurons of the AD cases (B); (C-D) Phosphorylated S1981 ATM (P-ATM) is much less prominent in control (C) than in AD (D) material; (E-F) Phosphorylated T68 of the cell cycle checkpoint kinase, Chk2 (P-Chk2) is less apparent in control (E) than in AD (F) material, but the cells that are stained in the AD deep nucleus are most likely astrocytes, not neurons. The insert in F validates this interpretation. Its shows double staining of P-Chk2 (red) and GFAP (green), an astrocyte marker.

Cellular stresses, including DNA damage, have been linked to cell cycle deregulation in neurons [31,32]. Our observation of cell cycle reactivation in neurons of the cerebellar dentate nucleus in AD prompted us to investigate the status of the DNA damage sensor and G2/M check point protein, 53BP1. 53BP1 is a p53 binding protein, which has been shown to be a reliable marker for DNA damage [33]. We found that there were many more 53BP1 stained cerebellar dentate nucleus neurons in AD brains (Figure 2B) than in controls (Figure 2A) and this difference was statistically significant (p = 0.001, Table 1). Further, the level of 53BP1 expression correlated well with AD diagnosis (p = 0.01, Table 2).

53BP1 is also a substrate of ATM (ataxia-telangiectasia mutated), a PI3 kinase that is activated by auto-phosphorylation (on serine 1981) soon after DNA double strand breaks appear. To determine whether the 53BP1 response was reflective of a broader ATM-dependent response to DNA damage, we examined ATM auto-phosphorylation with a phospho-ser1981 specific antibody (Figure 2C, D). We found significantly increased numbers of phospho-ser1981 ATM (P-ATM)-positive cerebellar dentate neurons in the AD cases compared to the control material (p = 0.0001) supporting the data we obtained from the 53BP1 immunostaining (Table 1 and Figure 2A, B). There was a good correlation between the two DNA damage makers (r = 0.6, p = 0.003). Three cases (2 AD, 1 control) had extensive P-ATM staining but were not immunoreactive to 53BP1 antibody. The biological implication of this discordance between P-ATM and 53BP1 in these cases is not clear. Overall, P-ATM seemed to correlate with AD diagnosis better than 53BP1 (Table 1 and 2). Of note, staining with phospho-specific antibodies against the phosphorylated isoform of the cell cycle checkpoint kinase, Chk2 (P-Chk2), was also positively correlated with disease state (Figure 2E, F), but the staining was not in the cerebellar dentate neurons themselves. Instead, activated P-Chk2 was observed in mostly small, non-neuronal cells. These cells were most likely activated astrocytes as they could be double labeled with GFAP and P-Chk2 (Figure 2F, inset).

### Onset of deep nuclear abnormalities in the Alzheimer's disease process

The finding of a strong disease correlation with several of the stress markers used in this study raises the question of whether the cerebellar dentate nuclear involvement is present from the beginning of the disease or is, instead, a later symptom of disease progression. To address this question, we stained five samples from individuals with very early signs of AD (CDR 0.5) (Table 3). Of the five, only two were significantly reactive for either cell cycle or DNA damage makers in cerebellar deep nuclei. Case HCB07 had significant numbers of 53BP1-positive CDN neurons as well as several PCNA positive cells, likely indications of DNA damage. We detected no cyclin A staining in this case (nor was there significant P-ATM staining). The reverse situation was found in case HCB11, which had significant numbers of cell cycle events but little evidence of a DNA damage response. This was in contrast to material from the hippocampus of the same cases. Here, as reported previously [22], robust cell cycle reentry was found in all 5. Markers of DNA damage were also elevated in this area. While the sample size is small, the strong suggestion is that cerebellar involvement begins after the disease has become manifest in neocortical and hippocampal structures.

**Table 3:** Cell cycle and DNA damage markers in early Alzheimer's disease

Cerebelar Nuclei				
**Case**	**Con/AD**	**Cyclin A**	**pATM1981**	**53BP1**

HCB03	AD(0.5)	0	0	0
HCB04	AD(0.5)	0	0	0
HCB07	AD(0.5)	0	0	3
HCB08	AD(0.5)	0	0	0
HCB11	AD(0.5)	5	1	0

				

**Hippocampus**				

**Case**	**Con/AD**	**Cyclin A**	**pATM1981**	**53BP1**

HCB03	AD(0.5)	2	4	3
HCB04	AD(0.5)	2	3	2
HCB07	AD(0.5)	1	4	3
HCB08	AD(0.5)	1	1	1
HCB11	AD(0.5)	1	4	5

Five cases of early AD from the Washington University at St. Louis ADRC were stained for the presence of cell cycle proteins and DNA damage markers in the cerebellar dentate nuclei and hippocampus. Each sample was scored based on % of CDN neurons stained and the staining intensity.

### Cell cycle deregulation and the DNA damage response

**Figure 3:**
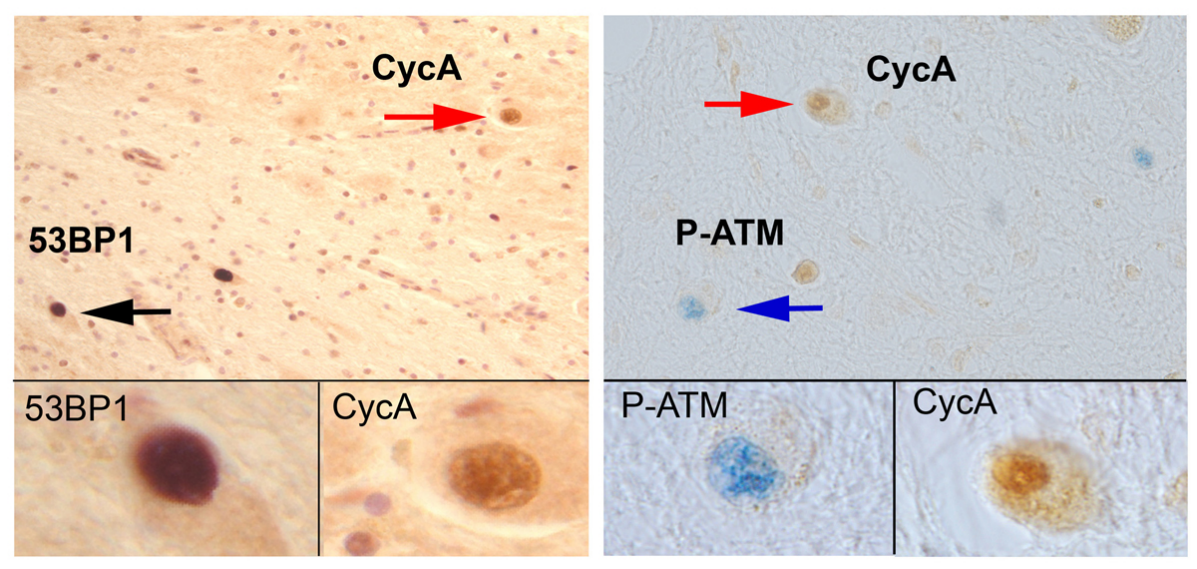
**Cyclin A expression negatively correlates with 53BP1 or P-ATM expression in cerebellar dentate nuclei**. Two sections from an AD patient were first stained with cell cycle marker Cyclin A (CycA), then stained with DNA damage markers 53BP1 or P-ATM. Staining of Cyclin A was visualized by peroxidase substrate DAB (brown staining, red arrow). 53BP1 (left panels) or P-ATM (right panels) was visualized by Vector VIP (purple staining, black arrow) or Vector blue (blue staining, blue arrow). Cyclin A and the DNA damage markers 53BP1 or P-ATM were consistently found in different CDN neurons, the insets represent high magnification reproductions of the cells indicated by the arrows in the main panel.

The fact that cell cycle and DNA damage events are not co-localized during early stage AD suggests that these two events are temporally sequential or mutually exclusive. This is somewhat unexpected as DNA damage has been shown to cause cell cycle reentry in cultured neurons [31,32]. It might also be presumed that forced cell cycle re-entry in a post-mitotic neuron would induce DNA damage. We re-examined our cerebellar data and found that, analyzed case by case, there was no significant correlation between the cell cycle and DNA damage markers we tested in this study. Within each case, a high cell cycle index (cyclin A) did not correlate with a high score in the DNA damage response (P-ATM) and vise versa (Table 1 and 3). To determine whether there is a link between DNA damage and cell cycle deregulations in individual cerebellar dentate neuron, we performed double immuno-labeling of the human AD sections. We found no overlap between the two processes in either cerebellum or hippocampus. An example of our findings from the CDN can be found in Figure 3. The cell cycle marker cyclin A (Figure 3 red arrow) and the DNA damage marker 53BP1 (Figure 3 black arrow) and P-ATM (Figure 3 blue arrow) were consistently found in different CDN neurons. Coupled with our observations from cases of early stage AD (Table 3), the findings suggest that DNA damage and cell cycle reentry are mutually exclusive events in the CDN and hippocampal pyramidal neurons.

### FISH (Fluorescent In Situ Hybridization) reveals hyperploidy in deep nuclear neurons

Elevated levels of cell cycle proteins are not normally found in post-mitotic nerve cells. When they appear, they are usually taken as evidence for the re-initiation of a cell cycle process. The discovery of two indicators of DNA damage in the same neuronal population, however, cautions that the proteins might be expressed in the service of DNA repair rather than DNA replication. To relate our findings with cell cycle and DNA damage proteins to the presence of true cell cycling, we examined the number of chromosome copies present in cerebellar dentate neurons of AD and control cases using fluorescent in situ hybridizaiton (FISH). A total of 16 samples (8 AD, 5 controls, 3 disease controls) proved usable for this analysis. The remaining cases were excluded because of technical difficulties related to the preservation of the autopsy material. These difficulties precluded us from doing FISH analysis, even though all samples were suitable for immunohistochemistry.

**Figure 4:**
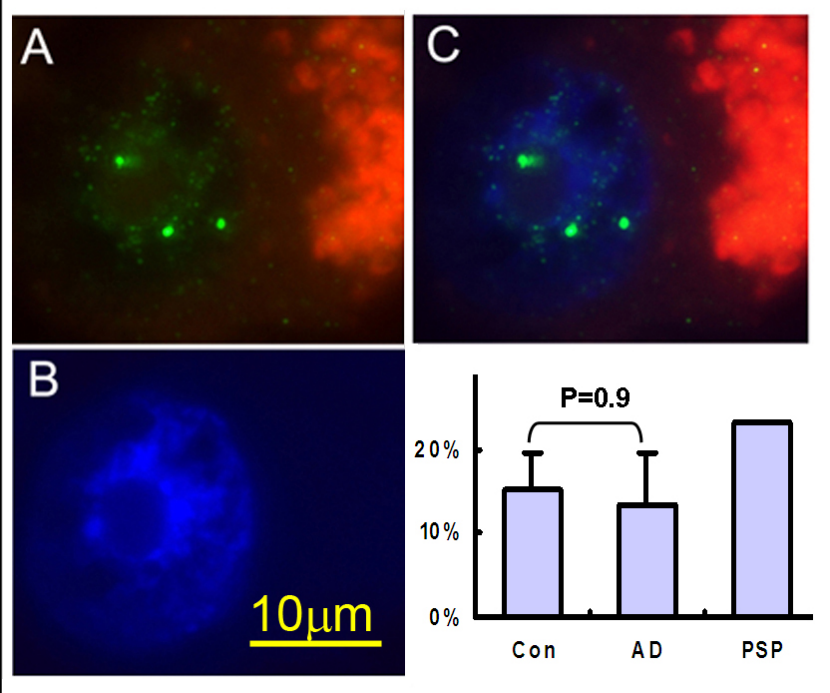
**The incidence of hyperploidy is high in dentate nucleus neurons from both AD and control samples**. Figures A-C show a representative polyploid cerebellar dentate nuclear neuron. (A) FISH probe fluorescence (green spots) indicates three genomic copies of the probe. The red color is due to the autofluorescence of the neuronal lipofuscin. (B) DAPI counterstain shows the location of the nucleus. (C) Merge of A and B. (D) Bar graph shows the results of scoring of about 100 neurons per case for the presence of 3-4 spots in a single dentate neuronal nucleus. The t-test statistic indicates that there is no significant difference (p = 0.9) between controls (15.2 ± 4.4%) and AD (13.5 ± 6.1%).

Unexpectedly, we found a very high rate of polyploidy (extra chromosome copies indicating DNA replication) in the neurons of the CDN in both AD and control samples. The values ranged from 4% to 23% of the total number of deep nuclear neurons (Figure 4). Averaged across all probes and all cases, we observed no significant difference (p = 0.9) in the percentage of hyperploid dentate nuclear neurons between AD (13.5 ± 6.1%) and controls (15.2 ± 4.4%). FISH evidence for high levels of hyperploidy in both AD and control samples have been documented before by other labs but these results are in apparent conflict with our previous report on human hippocampus material [21]. We discuss the reasons for this discrepancy in more detail in the Discussion section. We also scored 3 'disease controls'; two had been diagnosed with frontotemporal lobe dementia-17 (16.5% hyperploid neurons) and one with progressive supranuclear palsy (PSP, 23% hyperploid neurons).

**Figure 5:**
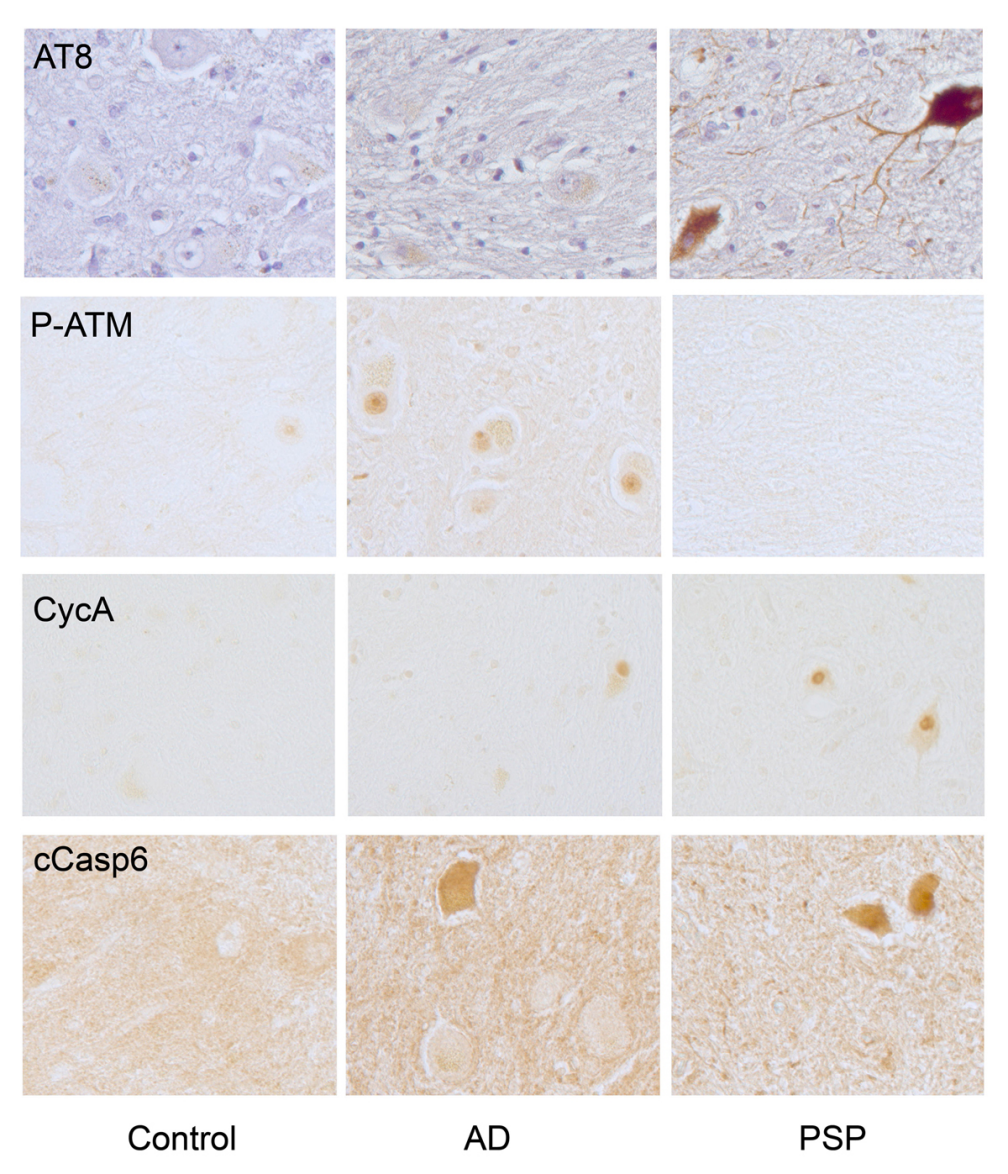
**A comparison of various neuronal stress markers in the CDN of AD, PSP (progressive supranuclear palsy) and control**. Top row: while hyperphosphorylated tau (AT8 staining) is absent in control (column 1) and AD (column 2) samples of CDN, its expression in PSP neurons (column 3) is quite strong. Second row: the reverse pattern is found for the DNA damage response (P-ATM). DNA damage is prevalent in AD but absent in PSP; control samples are negative. Third row: cell cycle reentry (CycA immunostaining) reveals yet a different pattern; it is absent in controls, but present in CDN neurons of both AD and PSP. Fourth row: the cell death marker, cleaved caspase 6 (cCasp6) appears to reproduce the pattern seen with the cell cycle markers; clear neuronal staining in both AD and PSP.

Though represented by only a single individual, the PSP case is particularly interesting. The high rate of polyploidy found in this case is consistent with the fact that PSP patients manifest cerebellar ataxia [34]. Intriguingly, this PSP case also had the highest percentage of CDN stained with cyclin A, a robust presence of the PHF-1 tau epitope, an increase in staining for the cell death marker, cleaved caspase 6, and a clear reduction of neuronal density (Figure 5). However, this case stained negative for DNA damage response markers 53BP1 and P-ATM. Taken together these results suggest an important link in this condition between true CCE and neuron loss.

### Clinical-pathological correlations

The appearance of a cellular stress response - DNA damage and cell cycle protein expression - in the cerebellar dentate neurons suggests that there might be detectable deficits in cerebellar related neurological signs. We retrospectively reviewed the neurological symptoms reported in the subjects' clinical records and attempted to correlate the reported symptoms with the presence or absence of cell cycle and DNA damage markers. This review was limited to any chart notations suggesting motor symptoms that might be cerebellar in origin - intention tremor, clumsiness or overt ataxia - and unorthodox cerebellar signs such as speech/language deficiencies and motor planning difficulties. None of the classic cerebellar signs correlated in any significant fashion with the DNA damage response, with cell cycle protein levels or with polyploidy in the deep cerebellar neurons. There was, however, a clear correlation between cyclin A expression and unorthodox cerebellar signs. Moderate to high levels of cyclin A in CDN seem to predict deficits in speech, language and motor planning, however, the reverse was not true (Table 4).

**Table 4:** Correlation of neurological symptoms with cell cycle and DNA damage markers

Case ID	Age	Gender	Diagnoses	Braak stage	CCE (CycA)	DDR (P-ATM)	Motor status	Unorthodox cerebellar signs		Classic cerebellar signs				
									
								*S/L*	*MP*	*Bal*	*AT*	*IT*	*NYS*	*WBS*
A06-149	81	F	AD REG	V/VI	++	+++	abnormal	yes	yes	yes	no	no	no	no
A08-14	81	M	AD REG	V/VI	+	+++	abnormal	yes	yes	yes	no	no	no	no
A08-15	86	F	AD REG	V/VI	+	++	abnormal	no	no	no	no	no	no	no
A08-32	75	F	AD REG	VI	+	++	normal	no	no	no	no	no	no	no
A08-42	35	M	AD REG	V/VI	+	+++	abnormal	no	yes	yes	no	no	no	no
A08-63	55	M	AD REG	VI	+	+++	abnormal	no	yes	yes	no	no	no	no
A08-75	90	F	AD REG	V	-	+++	normal	no	no	no	no	no	no	no
A08-78	76	M	AD REG	VI	++	+++	abnormal	yes	yes	yes	no	no	no	no
A08-52	85	F	PSP-R	SEVERE	+++	+	abnormal	yes	yes	yes	no	no	no	no
A08-19	52	M	GI BLEED	V	++	-	abnormal	yes	yes	no	no	no	no	no
A08-20	83	M	ETOH/CM	V	+	+++	normal	no	no	no	no	no	no	no
A06-127	74	M	AML/GVHD	0	-	-	normal	no	no	no	no	no	no	no
A08-13	77	F	CHF	I/II	-	+	abnormal	no	no	yes	no	no	no	no
A06-106	73	M	CYSTIC FIBROSIS	0	-	-	normal	no	no	no	no	no	no	no
A06-69	78	M	INFARCTS	0	-	-	abnormal	yes	yes	yes	no	no	no	no
A06-131	85	M	SEPSIS/CHF	0	-	-	abnormal	yes	no	yes	no	no	no	no

Sixteen subjects that had both clinical records of neurological symptoms as well as immunohistochemistry results for cyclin A and P-ATM are presented here. Each sample was scored based on % of CDN neurons stained and the staining intensity. "-" means no staining; "+" means very few cells stained or weakly stained; "++" means moderate staining and "+++" means robust staining in more than half of the CDN neurons counted. None of the classic cerebellar signs correlated with DNA damage response or cell cycle protein level. But, there was a clear correlation between high cyclin A expression in CDN and unorthodox cerebellar signs - speech/language and motor planning. Abbreviations: S/L (speech/language), MP (motor planning), Bal (balance), AT (ataxia), IT (intention tremor), NYS (Nystagmus) and WBS (wide base stance).

## Discussion

In this study, we provide evidence that cerebellar dentate neurons are targets of AD pathology. As the neurological and behavioral symptoms and neurobiological aspects of AD are complex, a distributed pathology that includes the cerebellum is not unexpected. We submit that our data argue for the inclusion of the cerebellar dentate nucleus in the list of nervous structures that are affected in AD.

It is intriguing to consider AD symptomatology in light of the non-motor functions of the cerebellum. Decreased verbal fluency is an early finding in AD, and may be related to retrieval slowing. The participation of the cerebellum in speech and language has been recognized for some time (Schmahmann and Sherman, 1998 [3] and references therein). Functional imaging studies show that the lateral cerebellum is engaged when a subject performs a "word generation" task such as determining an appropriate verb to use with a particular noun [35]. Planning and problem solving difficulties may also be related to the degradation of cerebellar function. Whether or not the cerebellum is primarily involved in the actual cognitive dimensions of these processes, decreased cerebellar function places an individual at a disadvantage when it comes to sequencing and planning complex tasks. There is also physiological evidence for anatomical connections between cerebellar and limbic structures [1]. Schmahmann and Sherman designate the totality of these symptoms as the "cerebellar cognitive affective syndrome" [3]. Our findings suggest that the full clinical syndrome of AD includes deficits that are due in part to loss of neuronal function in the deep cerebellar nuclei.

We are not the first to suggest cerebellar involvement in AD [6-11,36]. However, to our knowledge, there have been no reports that directly link the cerebellar deep nuclei to AD. Our findings also add a new type of evidence to the argument. We have shown that in AD a portion of the cerebellar dentate neurons manifest higher levels of markers associated with DNA damage and cell cycle events. If the appearance of the S-phase cyclin, cyclin A, is used as a marker there is a significant cell cycle involvement of the neurons of the deep cerebellar nuclei in AD cases compared to controls. The levels of cyclin A are significantly correlated with disease state. Paradoxically, however the appearance of elevated PCNA levels (a second S-phase marker of the cell cycle) are not disease correlated. The discrepancy may be explained by the fact that PCNA is involved in both cell cycle progression and DNA damage repair [30].

FISH evidence for high levels of hyperploidy (extra chromosomal material) in both AD and control samples raises several important issues. The absence of correlation between the appearance of cell cycle proteins and evidence of DNA replication might be due to the fact that expression of specific cell cycle proteins can be turned on and off. Polyploidy, by contrast, is irreversible. If the polyploid cells do not die, or if they die slowly, they would remain a substantial fraction of the population, even if regulation of cell cycle protein levels returned to normal. Perhaps more significant is the fact that the high levels of hyperploidy we observed in the non-demented control cases is at odds with our earlier studies in hippocampus and basal nuclei [21]. This discrepancy can be partly explained by the increased sensitivity of the method used in the current study compared to that used by us previously. Other labs have addressed the same problem [37-40]; the common thread in all reports is that while the absolute numbers of hyperploid neurons in normal individuals may be uncertain, this number is significantly increased in affected regions of the Alzheimer's disease brain. We also take note of recent lineage tracing studies showing that the deep cerebellar neurons are derivatives of the rhombic lip rather than the ventricular zone of the fourth ventricle [41]. As the rhombic lip is the source of one of the few known tumors of the CNS that is neuronal in origin (medulloblastoma) it is possible that cell cycle regulation in this lineage is unique among the neurons of the central nervous system.

The expression of DNA damage-related proteins in the deep cerebellar neurons is a robust and consistent marker of disease in the cerebellar dentate nucleus. The appearance of DNA damage during the course of late-onset neurodegenerative disease has been attributed in part to the fact that neurons exhibit high mitochondrial respiration, which is known to lead to the production of reactive-oxygen-species. Over time this oxidative stress results in the accumulated damage of mitochondrial and nuclear DNA [29]. In the current report we show that the chromosome binding protein, 53BP1, and the activated DNA damage response kinase, P-ATM, are both significantly elevated in AD cases with Braak stage of V or above. The occurrence of DNA damage in AD hippocampus has been reported previously using the modified histone, γ-H2AX as marker [42]. In this study, however, the response was restricted to astrocytes and discussed as representing a part of the reactive astrocytosis. In the current study, it is the neurons within the cerebellar dentate nucleus that show the robust DNA damage response. This discordance will be pursued in a future study, especially as we have shown that the Chk2 checkpoint kinase protein, a downstream substrate of ATM, is activated in the astrocyte population of the cerebellar dentate nuclei (in keeping with the findings of Myung et al. [42]).

The cases we were able to study of individuals who died at early stages of their dementia (CDR 0.5), while few in number, are particularly informative as to the onset of deep nuclear abnormalities in the Alzheimer's disease process. Of the 5 individuals we examined, only two had evidence of either DNA damage or cell cycle protein expression in CDN. This can be contrasted with the hippocampus data from the same individuals in this study (Table 3) and our earlier work in the entorhinal cortex and hippocampus [22]. In every case, hippocampal neuronal cell cycle events were found to be as prevalent in individuals with mild cognitive impairment (MCI) as they were in cases that had progressed to full AD. The implication is that the involvement of the cerebellar dentate nucleus occurs later in the progression of the disease. The fact that cell cycle and DNA damage events are found in two different cases of early stage AD also suggests that these two events are temporally sequential or mutually exclusive. Indeed, accumulating evidence has suggested a weak response to DNA damage is associated with the initiation of a quick death, and this correlation may underlie aspects of the selective neuronal vulnerability observed in aging and neurodegenerative disease. For example, the number of Purkinje cells is significantly reduced during aging, but there is no parallel increase in DNA damage. By contrast, granule cell loss is insignificant during the aging process, but there is a significant increase in DNA damage markers in these cells [43,44]. In this regard it is worth considering that P-ATM and 53BP1 immunoreactive neurons in cerebellar dentate nucleus might persist, while cyclin A positive neurons might be destined to die. This prediction is consistent with the clinical-pathological data that link high cyclin A expression to difficulty in speech/language and motor planning.

## Conclusions

The results presented here reveal that the CDN neurons are under cellular stress in AD and suggest that at least some of the non-motor symptoms found in patients with AD may be partly cerebellar in origin. Our findings also emphasize the value of using direct markers of neuronal distress - cell cycle events and DNA damage - as pathological markers in AD. They augment the classical histopathological picture achieved by staining for amyloid plaques and tau inclusions by providing an early neuronal vulnerability marker. As with the appearance of hyperphosphorylated tau, increased neuronal cell cycle events are not disease specific [23]. However, they unify the disease phenotype in human AD and its transgenic mouse models, and provide a perspective on the human disease that adds depth and insight.

## Methods

### Human cases

Formalin-fixed and paraffin-embedded 10 μm consecutive sections from cerebellar cortex and dentate nuclei from 46 AD and age-matched controls were obtained from the Alzheimer's Disease Research Centers at Case Western Reserve University and Washington University School of Medicine. Some of the cases we used for the study had been clinically diagnosed with AD and were subsequently confirmed with standard pathological examination. Other cases died without a clinical diagnosis of AD, but, neuropathological examinations were scored as being either Braak stage V or VI. For the purposes of this study these cases were defined as AD. In addition, 5 cases of early stage AD (CDR0.5), obtained from the Washington University Alzheimer's Disease Research Center, were also included in the study. The Clinical Dementia Rating (CDR) [45] reflects cognitive status at the neurological exam most immediately preceding death. The CDR scale ranges from 0 to 3 (0, 0.5, 1, 2, and 3). A CDR score of 0 indicates no dementia.

### Antibodies

All antibodies used here are commercially available. To detect DNA damage, we used a rabbit polyclonal 53BP1 antibody (ab36823, Abcam); a rabbit polyclonal antibody against phosphorylated Chk2 kinase (on threonine residue 68) - P-Chk2 (ab38461, Abcam) and a rabbit monoclonal antibody against phosphorylated ATM kinase (on serine residue 1981) - P-ATM (ab81292, Abcam). The specificity of these antibodies was confirmed by Western blot and by immunofluorescent staining in neuroblastoma cells treated with etoposide and H_2_O_2_. Antibodies against cyclin A (ab7956, Abcam), Cdk4 (sc-260, Santa Cruz Biotech), and PCNA (ab18197, Abcam; sc-56, Santa Cruz Biotech) were used to detect cell cycle events. Cell death was evaluated using cleaved caspase 6 antibody (ab52295, Abcam). Phosphorylated tau monoclonal antibody (AT8) and beta amyloid, 1-16 monoclonal antibody (6E10) was purchased from Thermo Scientific and Covance. The specificity of these antibodies has been established previously and confirmed by us through the use of Western blots.

### Immunohistochemistry

The human materials were formalin-fixed, paraffin-embedded and sectioned at 10 μm sections. These were treated as described previously [20]. All sections underwent antigen retrieval with high temperature citrate buffer for 20 min, and then soaked in 0.3% hydrogen peroxide to remove endogenous peroxidase activity. Primary antibody was diluted in 10% goat serum with 0.5% Tween-20. The primary antibodies used and their dilutions were as follows: 53BP1, 1:1000; P-ATM, 1:250; P-Chk2, 1:250; Cyclin A, 1:500; Cdk4, 1:200 and PCNA, 1:2000. Primary antibodies were detected using biotinylated goat anti-rabbit or anti-mouse secondary antibody (1:400), avidin-biotin complex horseradish peroxidase and peroxidase substrates including DAB, DAB+Ni and VIP (Vector Laboratories). Some sections were counter-stained with hematoxylin QS. For double staining, alkaline phosphatase linked secondary antibodies and Vector Blue or Vector Red were used (Vector Laboratories). Stained sections were photographed and viewed at a final magnification of 200 using Leica Application Suite/Leica DM5000B. All neurons in the serpentine belt of cerebellar dentate nuclei on each section were examined. Each case/section was given a score according to the following criteria: 0 = no staining; 1 = very few cells stained or weakly stained; 2 = 5-10%, moderately stained; 3 = 10-25% moderately to strongly stained; 4 = more than 25% strongly stained; 5 = more than 50% strongly stained.

### Fluorescent in situ hybridization (FISH)

FISH was performed as described previously [21]. Briefly, chromosome 11 probe (11q23.3-24.1) was made from a bacterial artificial chromosome (BAC), which encodes the human BACE1 gene. BAC DNA was labeled by standard nick-translation protocols using digoxygenin-labeled dUTP (Roche). Sections were deparaffinized, pretreated with 30% Pretreatment Powder (Oncor) for 11-15 min at 45°C, and digested with 0.25 mg/ml protease in 2 × SSC (Oncor) for 20-25 min at 45°C. For each reaction, 100 ng of probe was applied to each section and denatured with the sections at 90°C for 12 min. The sections were then incubated at 37°C overnight. The next day, slides were washed with 50% fomamide/2 × SSC, 0.1 × SSC and then PBST. Primary and secondary antibodies used to detect digoxygenin-labeled DNA were: mouse anti-digoxygenin (Vector Laboratories) and Alexa 647 goat anti-mouse (Invitrogen) both diluted 1:200 in 10% goat serum. Sections were counterstained with DAPI (Invitrogen). An average of 100 large cerebellar dentate nucleus neurons per section was scored. AD and control cases were processed under the identical conditions.

### Statistics

Control cases with Braak's stage V and higher were grouped with AD for statistical analysis. Statistical significance was calculated using Student's t-test, Fisher's exact test and Chi-square with Yates correction. Correlation (r = Multiple R) and p values for the null hypothesis (no correlation) were calculated using Excel Data Analysis Tool/Regression.

## Competing interests

The authors declare that they have no competing interests.

## Authors' contributions

KH and JC conceived and designed the experiments. JC performed the immunohistochemistry and fluorescent in situ hybridization analysis. MLC assisted by YY identified and prepared the cerebella paraffin sections. AJL provided the neurological evaluation. All were involved in the preparation of the manuscript and approved the final version.
